# HEV in Blood Donors in Switzerland: The Route to Safe Blood Products

**DOI:** 10.3390/pathogens13100911

**Published:** 2024-10-18

**Authors:** Mauro Serricchio, Peter Gowland, Nadja Widmer, Martin Stolz, Christoph Niederhauser

**Affiliations:** 1 Interregional Blood Transfusion SRC, 3008 Bern, Switzerland; mauro.serricchio@itransfusion.ch (M.S.); peter.gowland@itransfusion.ch (P.G.); martin.stolz@itransfusion.ch (M.S.); 2 Institute for Infectious Diseases, University of Bern, 3012 Bern, Switzerland

**Keywords:** hepatitis E virus, HEV, blood donors, infectious disease, seroprevalence, epidemiology, Switzerland, genotype, transfusion safety, surveillance

## Abstract

The hepatitis E virus (HEV) is an emerging infectious disease with zoonotic potential, causing acute hepatitis in humans. Infections in healthy individuals are often acute, self-limiting and asymptomatic, thus leading to the underdiagnosis of HEV infections. Asymptomatic HEV infections pose a problem for blood transfusion safety by increasing the risk for transfusion-transmitted HEV infections. Here, we describe the journey from determining the HEV seroprevalence among blood donors to the implementation of routine HEV RNA testing of all blood products in Switzerland in 2018 and summarise the HEV cases detected since. In total, 290 HEV-positive blood donations were detected by mini-pool nucleic acid testing (NAT) in Switzerland in the period of October 2018–December 2023, equal to an incidence of 20.7 per 100,000 donations. Thanks to the implemented scheme, no transfusion-transmitted infections occurred in this period. Furthermore, blood donation monitoring has proven to be an effective means of detecting HEV outbreaks in the general population. HEV cases in Swiss blood donors are caused by two major genotypes, the Swiss-endemic subtypes 3h3 and 3c. Interestingly, 11 HEV cases (5%) were of genotype 3ra, a variant found in wild and farmed rabbits. Our results indicate that mini-pool NAT is an efficient method to reduce the risk of transfusion-transmitted HEV infections.

## 1. Introduction

Hepatitis E virus (HEV), formally known as *Paslahepevirus balayani*, is an emerging infectious disease and the main cause of acute viral hepatitis, with an estimated 20 million HEV infections worldwide that lead to 44,000 deaths per year [1,2]. The HEV was initially isolated in 1978 as a causative agent of large endemic outbreaks of non-A and non-B hepatitis [3,4]. The single-stranded RNA virus belongs to the species Orthohepevirus A. The eight distinct genotypes reported infect a variety of mammals, and genotypes 1–4 and 7 are known to infect humans [2]. Human-infective HEV genotypes 1 and 2 are transmitted faeco-orally, most commonly through contaminated water sources, whereas genotypes 3 and 4 have a zoonotic origin and are transmitted mostly through the consumption of undercooked pork meat or cured meat products [5,6,7].

Chronic diseases with clinical persistence of the virus can occur in immunocompromised individuals [8]. Common symptoms observed in HEV patients are similar to other types of acute viral hepatitis and include jaundice, nausea, vomiting, fever and abdominal pain [2,9].

Since the mid-2000s, several studies have documented HEV transmission via contaminated blood products and organs in different European countries [10,11,12,13,14,15,16,17,18,19,20]. Many HEV transfusion-transmitted infections are asymptomatic, but serious chronic infections can occur in immunocompromised patients or in transplant recipients. The extensive travel habits and the consumption of various kinds of potentially contaminated cured meat products in our donor cohort make HEV transmission a potential threat to blood recipients and pose a challenge for blood safety [21,22,23].

In this paper, we describe the decision path regarding safe blood products in relation to the hepatitis E virus in Switzerland. A seroprevalence study in Swiss blood donors was carried out as the basis for the initial decision. In addition, data from the literature on anti-HEV seroprevalence in Europe and data concerning transfusion-transmitted cases were compiled in order to decide for or against the introduction of the HEV NAT screening in Switzerland. In the second step, the data from the first two years of screening were analysed to make a definitive decision for or against the implementation. Finally, the data compiled in relation to incidences, viral loads and genotypes found over the last 5 years are now presented.

## 2. Materials and Methods

### 2.1. Data Collection for Decision-Making

Before a decision was made by the national Blood Transfusion Switzerland of the Swiss Red Cross (BTS SRC) on whether to introduce temporary HEV-NAT screening in Switzerland, seroprevalence and incidence data, as well as data on transfusion-transmitted cases from Europe, were compiled. For this, a publication search in the National Library of Medicine was performed (https://pubmed.ncbi.nlm.nih.gov/). In addition, an HEV seroprevalence study was conducted in blood donors in Switzerland.

### 2.2. Study Population and HEV RNA Detection and Characterisation

Whole blood and apheresis donations were collected between 1 October 2018 and 31 December 2023 at regional Swiss blood transfusion services and tested for HEV RNA in one of five regional blood donation test centres. Two commercial HEV RNA assays were used: cobas HEV on the 6800/8800 platforms (Roche Diagnostics, Rotkreuz, Switzerland) and the Procleix HEV assay on the Procleix Panther platform (Grifols Diagnostic Solutions, Emeryville, CA, USA). Mini-pools of 16 to 24 donations were analysed with the cobas HEV assay and mini-pools of 12 to 15 donations were analysed with the Procleix HEV test. The cobas HEV RT-PCR test has a reported 95% of limit of detection of 18.6 IU/mL, whereas the Procleix HEV transcription-mediated amplification (TMA) assay has a reported 95% of limit of detection of 7.9 IU/mL. The sensitivity limits in an individual donation with the used assays and their corresponding mini-pools were thus as follows: cobas HEV to pools of 16 donations (298 IU/mL) and pools of 24 donations (446 IU/mL); and the Procleix HEV assay to pools of 12 donations (96 IU/mL) and pools of 15 donations (120 IU/mL). Reactive pools were resolved to the individual donation containing HEV RNA, with the same assay used for the pool screening. All reactive donations were subjected to confirmation testing at the national Swiss blood donor reference laboratory for infectious diseases. The viral load was determined with the RealStar HEV RT-PCR assay (Altona Diagnostics GmbH, Hamburg, Germany). The in-house-determined lower limit of detection (95% cut-off) of this assay was 5.0 IU/mL, as determined using a calibration curve based on the first World Health Organization International Standard for Hepatitis E Virus RNA Nucleic Acid Amplification Techniques (NAT)-Based Assays (PEI code 6329/10; https://www.pei.de). HEV serology was performed using the Wantai HEV IgG and IgM detection assays according to the manufacturer’s instructions (Eurobio, Les Ulis, France).

### 2.3. Phylogenetic Analysis

Sequence analyses were performed as previously described [24]. Viral RNA from 800 µL of plasma was extracted using the Qiagen Virus BioRobot 9604 kit (Qiagen, Hombrechtikon, Switzerland), modified for a Tecan Genesis platform (Tecan AG, Männedorf, Switzerland), and eluted in 80 µL of Qiagen elution buffer AVE. Viral cDNA was generated using random hexamers and Superscript IV (Thermo Fischer Scientific, Reinach, Switzerland) according to the manufacturer’s instructions. Nested PCR using HotStarTaq DNA Polymerase (Qiagen, Hombrechtikon, Switzerland) was performed to create a 493 nucleotide product of the 5′ region of the ORF2 (position 6029 to 6521 of reference genome AB630970) [25]. An alternative RT-PCR was conducted on some templates, producing a 589 nucleotide ORF2 product [26] (position 5752 to 6340 of reference genome AB630970). The forward and reverse primers used sanger sequencing (Mycrosynth GmbH, Balgach, Switzerland). To determine the HEV genotype, sequences were submitted to the online HEVnet typing tool (https://www.rivm.nl/mpf/typingtool/hev/ (accessed on 15 October 2024)). A 312 nucleotide overlapping sequence (position 6029 to 6340 of reference genome AB630970) was phylogenetically analysed using HEV reference strains [27,28]. Sequences were aligned using MUSCLE, and a Maximum Likelihood Tree with 1000 bootstrap replicates was created using the General Time Reversible model [29] (MEGA X software, version 10.1.7).

## 3. Results

### 3.1. Data Compilation from Europe and Seroprevalence Study of Blood Donors in Switzerland

Studies of HEV seroprevalences in the general population and among blood donors in different European countries have shown heterogeneous (from <5% to >50%) prevalence levels of anti-HEV IgG (Table 1).

Data gathered by blood donation services across Europe in the years 2012–2015 revealed significant HEV incidences, which ranged from 1:600 to 1:15,000 (Table 2) [11,12,18,38].

These observations prompted the Interregional Blood Transfusion Swiss Red Cross (IRB SRC) to conduct an HEV seroprevalence study during 2014–2016 with the blood of healthy donors from ten regional blood transfusion services of Switzerland. The results revealed that the overall HEV seroprevalence in Switzerland was 20.4%, with a significant variation in different Swiss geographical regions [43]. HEV IgG seropositivity ranged from 12.8% to 33.6%, with the canton of Geneva showing the lowest and the south canton of Ticino the highest prevalence. Anti-HEV IgG seroprevalence was lowest in young donors and increased with age [43]. A retrospective analysis within the canton of Bern revealed a decline of anti-HEV IgG seroprevalence over two decades, from 30.3% (1997/1998) to 27% (2006) to 22.3% in 2015/2016 [43].

A follow-up study focusing on the prevalence of anti-HEV antibodies in blood donors from the southern canton of Ticino confirmed the high anti-HEV IgG prevalences found in the previous study (32.8% (2012/2013) and 31.1% (2017–2019)) [44]. Regional differences within Ticino were detected, with rural and urban areas showing remarkable differences in HEV IgG positivity: 38.9% in the rural north versus 29% in the more urban south. In a quantitative risk assessment, the total burden of human foodborne hepatitis E in Switzerland from products containing pork liver was estimated to be 1481 cases per year in 2016 [45].

### 3.2. Implementation of Swiss HEV Testing of Blood Donors

Because of these high prevalences and disease burdens detected throughout Europe and Switzerland, a provisional mandatory blood donation NAT screening for HEV RNA was introduced in Switzerland, starting in October 2018. The required detection limit in an individual donation was set to 450 HEV IU/mL, and pools of 24 or less are currently being screened for HEV. In the following section, we are going to discuss the cases detected in Switzerland since the introduction of mandatory HEV testing.

### 3.3. Two-Year Monitoring

As predicted by the BTS SRC, a decision was made after two years of HEV-NAT screening as to whether this testing should be continued or not. In this time period, 541,349 blood donations were screened, and 125 positive donations were identified, showing an incidence of 1:4331 donations. At the time of blood donation, the HEV RNA-positive individuals were symptom-free. Their median viral load was 554 IU/mL (range: 2.01–2,500,000 IU/mL). Men (88; 70%) were more frequently infected than women (37; 30%) when compared with the sex distribution in the Swiss donor population (57% male/43% female) [24]. Based on the data obtained during these first two years, universal HEV RNA screening of all Swiss blood donations in mini-pools became mandatory.

### 3.4. Incidences Since Introduction of Mandatory HEV NAT

**Figure 1:**
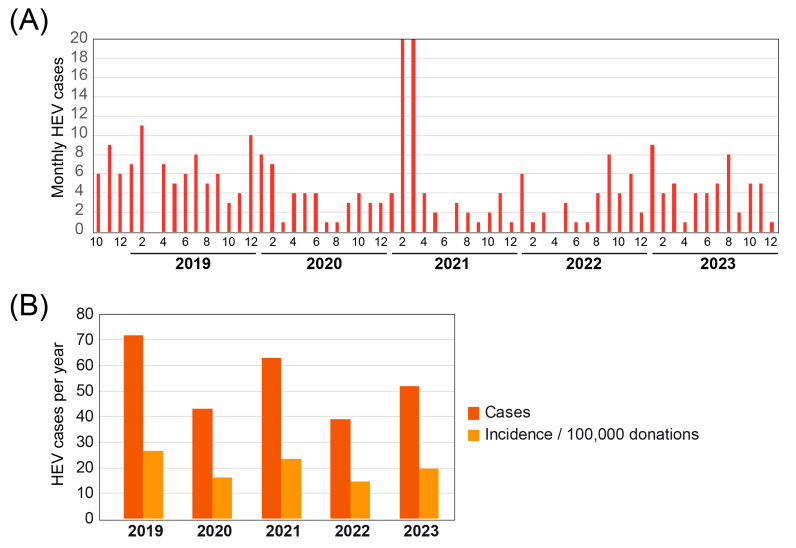
Summary of positive HEV cases detected since the introduction of mandatory mini-pool NAT in Switzerland in October 2018. (**A**) Monthly and (**B**) yearly HEV cases with the calculated incidence per 100,000 donations.

From the introduction of mini-pool NAT in October 2018 to the end of 2023, a total of 1,402,669 blood donations have been screened in Switzerland. Of these, 290 (0.02%) tested positive for HEV. This incidence was calculated to be 20.7 per 100,000 blood donations in the 5-year period. On average, we detected 55 positive HEV cases per year (Figure 1).

A sudden, steep rise in positive HEV cases in donated blood was detected at the beginning of 2021. The BTS SRC reported this significant increase to the Federal Office of Public Health (FOPH). A sharp increase in HEV reports was then also registered throughout Switzerland for patients for the same period, from January to May 2021. A total of 105 cases were reported during this period, 50 of which were positive blood donors (Figure 1) [1]. Compared to the previous three years, the number of notifications almost tripled. Men were predominantly affected, accounting for 64 per cent of those infected. The age distribution ranged from 18 to 87 years, with the average age of those infected being 54 years. Just under half of the reports (48%) were made following blood donation tests. The cases were spread throughout Switzerland. The FOPH then attempted to investigate the possible transmission routes. Unfortunately, no single cause of the outbreak could be identified [1]. Nevertheless, this example emphasises the importance of blood donor screening for monitoring ongoing infectious disease outbreaks.

### 3.5. Viral Loads, Genotypes and Serology

**Figure 2:**
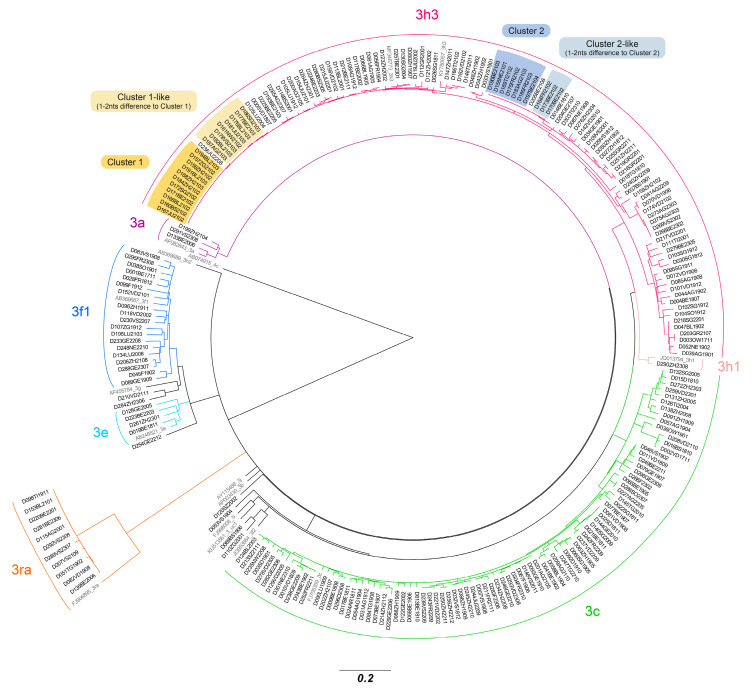
Phylogenetic analysis of HEV cases detected in Switzerland. A number of 312-nucleotide-long HEV sequences from positive HEV donations were phylogenetically analysed using HEV reference strains (grey). Sequences were aligned using MUSCLE and a Maximum Likelihood Tree with 1000 bootstrap replicates was created. Highlighted are two clusters (Cluster 1, yellow, and Cluster 2, blue) representing HEV cases from the outbreak from February/March 2021.

The Swiss reference laboratory for infectious disease markers analysed all positive blood donor HEV cases for confirmation and sequenced part of the viral genome. Viral load determinations revealed HEV titres that ranged from 1 IU/mL to 6 × 10^6^ IU/mL, with 55% of all cases having titres below 10^3^ IU/mL (Table 3). The median titre was calculated to be 717 IU/mL. Sequencing a fragment of the ORF2 allowed for the determination of the HEV genotype in 235 cases [24]. All of these were of genotype 3: 109 were caused by the Swiss-endemic HEV genotype 3h3 (49%) [46], 79 by 3c (33%), 18 by 3f1 (8%) and 11 by 3ra (5%) (Table 4). Genotype 3h3 was solely responsible for all the HEV cases detected during the outbreak from January to May 2021. A number of 312-nucleotide-long sequences of ORF2 were analysed using different reference strains and a phylogenetic tree was constructed. The results show that the cases of the outbreak form two distinct 3h3 clusters, with sub-clusters that differ in but 1–2 nucleotides (Figure 2).

In addition to genotyping, the Swiss reference laboratory also collected serological data on the presence of IgM and IgG anti-HEV antibodies in all positive HEV donors. Of all the 290 cases detected, 193 (67%) were IgM/IgG negative (−/−), 12 (4%) were IgM positive (+/−), 21 (7%) were IgG positive (−/+) and the remaining 64 (22%) were IgM/IgG positive (+/+) (Table 5). These data were in accordance with data obtained in the two-year survey [24].

## 4. Discussion

HEV is one of the leading causes of acute viral hepatitis worldwide [47]. In the European Union (EU), cases of hepatitis E have been increasingly reported over the last two decades [48]. Food-borne HEV genotype 3 infections predominate in developed regions [7]. Although acute HEV infections are typically self-limiting and sub-clinical and blood donors are feeling healthy at the time of donation, HEV infections pose a significant risk to recipients with compromised immune systems because they can develop a persistent HEV infection, with rapid progression to cirrhosis, decompensation and even death [49]. A retrospective study showed that the transfusion of contaminated blood products led to transfusion-transmitted HEV infections in 42% of recipients [13]. In Europe, based on studies published during the 2010s, the highest anti-HEV IgG prevalence levels were found in France (22.4%; with some regional prevalences > 50%), Poland (43.5%) and the Netherlands (27% and 31%) [30,32,50,51]. Prevalences between 10% and 20% were reported in other countries such as Austria, Denmark, Norway, Spain and the United Kingdom (England and North Wales) [34,35,37,52,53,54]. Ireland and Scotland showed proportions lower than 10% [42,55]. This variability in HEV prevalence was attributed to the studies’ geographical location, the included population, food production and eating habits, preferences for certain foods, contact with animals in rural areas [44], but also mainly to the performance characteristics of the anti-HEV IgG assay and the different test schemes used (individual or mini-pool).

The HEV seroprevalence in the blood donor population in Switzerland was shown to be rather high, with a value of 20.4% between 2014 and 2016 [43]. Based on these seroprevalence data, many European countries then considered whether to implement NAT HEV RNA screening as an additional safety measure.

Subsequently, HEV RNA incidences were also published from various countries. Very large differences were found here, depending on the production of food containing pork products, eating habits and the sensitivities of the RNA tests used in relation to the individual donation (4, 5, 11, 19). Based on the prevalence and incidence data observed in Europe and the relatively high seroprevalence in the Swiss donor population, mandatory nationwide HEV RNA testing was provisionally introduced for all blood donations in Switzerland in October 2018 (20). It was then decided that a two-year nationwide incidence study should be carried out for later evaluation of its definitive introduction by the National Blood Transfusion Service of the Swiss Red Cross.

This two-year study, initiated on 1 October 2018, analysed a total of 541,349 Swiss whole-blood and apheresis-blood donations, of which 125 donations were identified as HEV RNA-positive, corresponding to an incidence of 1:4331 donations (0.023%) [24]. The risk assessments in Switzerland, considering both the HEV seroprevalence and the HEV RNA incidence rate, led to the decision of a definite introduction of mandatory HEV RNA screening of all Swiss blood donations in the summer of 2022.

In the five-year period since the introduction of HEV NAT, HEV RNA has been confirmed in 290 out of 1,402,669 blood donations, corresponding to an overall incidence of 1 in 4837 donations (0.02%). When comparing the incidences after 2 and 5 years, it was noticeable that they were more or less at the same level. After 2 years, the incidence was found to be 1:4331 [24]; after 5 years it was 1:4837. All of the HEV RNA-positive donations from asymptomatic donors were successfully eliminated from the Swiss blood supply.

Of the 290 isolated HEV cases, 159 (55%) showed viral loads below 1000 IU/mL, and in 131 (45%) of the cases the viral loads were above 1000 IU/mL (Table 3). These viral load data are more or less on the same level as in the first two years of monitoring (57%/43%) but also in conformity with investigations performed in other countries such as Great Britain, France, Ireland or Spain [40,42,56,57]. Indeed, the viral loads found were between below 10 IU/mL and up to 10^6^ IU/mL; especially in the low range, these numbers depend on the sensitivity of the NAT system used.

The NAT sensitivity required for blood screening has been debated [58,59,60]. It is undeniable that a NAT system with high sensitivity, such as the one in Japan (<5 IU/mL), is needed to eliminate the transfusion transmission of HEV (TT-HEV). On the other hand, the majority of TT-HEV cases were caused by components containing >10^4^ IU HEV RNA [58,60]. If NAT with a sensitivity of 500 IU/mL (95% LOD) for a single sample is implemented, 93% of TT-HEV would be prevented in the setting of RBC transfusions [58,61]. If a component with 200 mL of plasma is considered, a sensitivity of 50 IU/mL would be required. Therefore, we must reconsider the optimal NAT sensitivity when TT-HEV cases with serious clinical outcomes accumulate, especially in relation to blood components containing very low viral loads. Over the last few years, the trend was that pooled NAT shifted towards smaller pool sizes or even ID-NAT, thus increasing the sensitivity of individual tests [62]. For HEV, the introduction of ID-NAT would further increase the safety of blood products and minimise TT-HEV infections. In facilities currently performing ID-NAT, the direct implementation of multiplex NAT, including HEV, could be advisable, as has been shown in Spain and Croatia [63,64].

It was possible to determine a genotype for 235 (81%) of the 290 positive samples. All cases belonged to genotype 3 (Table 4, Figure 2). Most virus strains clustered around two distinct HEV sub-genotypes: 3h3 (formerly 3h-s (p)), a variant endemic in the Swiss pig population and which is practically confined to Switzerland [46], and 3c, a variant often encountered in northern Europe. The remaining sub-genotypes identified are all known to circulate in Europe. Eleven 3ra sub-genotypes were identified, a variant associated with rabbits (Table 4, Figure 2).

As shown in Figure 1A, an HEV outbreak occurred in Switzerland between January and March 2021. Thanks to the rapid response of the blood transfusion services and the cooperation of several institutions, such as the Federal Office of Public Health, it was possible to investigate this outbreak in more detail. Although no contaminated food could be identified that triggered the outbreak, this outbreak investigation serves as an instructive example of good interdisciplinary collaboration between authorities, researchers and producers in the sense of the One Health approach. In that context, it is clear that surveillance data containing molecular information on circulating hepatitis E viruses in humans and animals across Switzerland will support the animal health and food safety authorities responsible for preventive measures in the animal population and food production.

None of the infected donors showed signs of acute or chronic HEV symptoms. Overall, 67% of the HEV RNA-positive donors were seronegative (IgM and IgG) at the time of donation and were thought to be in the early stages of infection. Some were reactive for only anti-HEV IgG antibodies without IgM, reflecting late infections (7%). In total, 22% were IgM- and IgG-positive, whereas only 4% were IgM positive (Table 5). These data have remained stable over the last 3 years (2020 to 2023) compared to the first 2 years (2018–2020) [24].

Here, we demonstrated how epidemiological data were used to develop a sensible measure to increase the safety of blood products in Switzerland. It has become clear that the blood donation system can play an important role in the management of epidemics thanks to its well-developed infrastructure and good knowledge of its blood donors.

## Figures and Tables

**Table 1:** HEV seroprevalences from blood donors in different European countries.

Country	HEV IgG Prevalence	Reference
France	52.2%	[30]
Germany	29.5%	[31]
The Netherlands	27.0%	[32]
England	12.0%	[33]
	16.0%	[34]
Austria	13.5%	[35]
Scotland	4.7%	[36]
Spain (Catalonia)	10.72–19.96% *	[37]

* Two different enzyme immunoassays (EIAs).

**Table 2:** HEV incidences in blood donors from different European countries.

Country	HEV Incidences	Reference
Sweden	1:7986	[11]
Germany	1:1240–1:6925	[11,12,39]
Great Britain	1:2848–1:14,520	[13,33,36]
France	1:2218	[40]
Austria	1:8416	[35]
Spain	1:3333	[37]
The Netherlands	1:611	[41]
	1:1440	[38]
	1:2700	[32]
Ireland	1:5000	[42]

**Table 3:** Viral loads of positive HEV plasma samples, quantified by RT-PCR.

Viral Load IU/mL	Cases	%	
1 to 10	15	5	55
11–100	43	15	
101–1000	101	35	
1001–10,000	73	25	45
10,001–100,000	40	14	
100,001–1,000,000	13	4	
1,000,001–10,000,000	5	2	
Total	290		

**Table 4:** HEV genotypes detected in HEV RNA-positive donations. Analysis of a partial ORF-2 DNA sequence shows a dominance of genotype 3 in the Swiss population. The 3h3 is a Swiss-endemic strain circulating in Swiss pig and wild boar populations.

Genotype	Cases	%
3h3	109	49
3c	79	33
3f1	18	8
3ra	11	5
3a	3	1
3e	4	2
3h1	1	
3 others	10	2
Total	235	

**Table 5:** Serological data of HEV-positive blood donations. Numbers of IgM- and IgG-positive or -negative blood samples from HEV-positive donations are shown (percentage of total in parentheses).

IgM/IgG −/−	IgM/IgG −/+	IgM/IgG +/−	IgM/IgG +/+
193	21	12	64
(67%)	(7%)	(4%)	(22%)

## Data Availability

The sequence data presented in the study are openly available from the National Center for Biotechnology Information at https://www.ncbi.nlm.nih.gov/, with accession numbers OV843868-OV843886, OZ077043-OZ077123, OV844702-OV844807 and OV844636-OV844673.
